# Pulmonary Mucosa‐Associated Lymphoid Tissue (MALT) Lymphoma: A Diagnostic Dilemma

**DOI:** 10.1002/rcr2.70386

**Published:** 2025-10-28

**Authors:** Rajan Patel, Diane Stover

**Affiliations:** ^1^ Department of Medicine, Pulmonary Service Memorial Sloan Kettering Cancer Center New York New York USA

**Keywords:** autoimmune disease, indolent lymphoma, MALT lymphoma, primary pulmonary lymphoma, rituximab

## Abstract

Primary pulmonary lymphoma (PPL) is a rare clonal proliferation of lymphoid tissue confined to the lungs, representing only 0.5%–1% of all lung neoplasms. Pulmonary mucosa‐associated lymphoid tissue (MALT) lymphoma is the most common subtype and is often associated with chronic inflammation and autoimmune conditions. Diagnosis is challenging given its indolent course, nonspecific clinical features and variable radiographic presentations. We report a 64‐year‐old female with systemic lupus erythematosus who was incidentally found to have multiple pulmonary nodules. Serial imaging revealed interval growth and biopsy confirmed pulmonary MALT lymphoma. A watchful waiting strategy was initially employed, but subsequent disease progression prompted rituximab therapy, with only mild radiographic response despite preserved clinical stability. This case highlights the diagnostic challenges of pulmonary MALT lymphoma, the variability in treatment response and the potential interplay with autoimmune disease. Further studies are needed to clarify optimal therapeutic approaches, including the role of macrolides and autoimmune‐directed therapies.

## Graphical abstract

This case report describes a patient with systemic lupus erythematosus who was incidentally diagnosed with pulmonary MALT lymphoma after years of indolent radiographic progression. Management included a prolonged period of observation followed by rituximab, with only a partial radiographic response. The case emphasises the diagnostic challenges and variable treatment outcomes of this rare lymphoma.

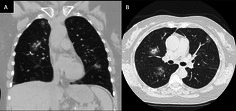

## 1 Introduction

Primary pulmonary lymphoma (PPL) is a rare clonal proliferation of lymphoid tissue confined to one or both lungs without evidence of extrapulmonary involvement at diagnosis. The incidence of PPL is very low, accounting for 0.5%–1.0% of all lung neoplasms. Amongst these, pulmonary mucosa‐associated lymphoid tissue (MALT) lymphomas account for majority of cases. MALT lymphoma is an extranodal B‐cell lymphoma originating from the mucosal layers of various organs such as the gastrointestinal tract, thyroid, salivary gland and less frequently, the lung. MALT lymphoma has been associated with chronic inflammatory states including those related to infection, smoking and autoimmune conditions. Unlike gastric MALT lymphoma, which is strongly linked to *Helicobacter pylori* infection, no definitive infectious aetiology has been established for pulmonary MALT lymphoma [1]. However, approximately 16% of pulmonary MALT lymphoma cases are associated with autoimmune disorders. Pulmonary MALT lymphoma typically presents without specific manifestations, has an indolent course and has substantial potential for misdiagnosis. This report presents a rare case of pulmonary MALT lymphoma and highlights the diagnostic challenges in this disease.

## 2 Case Report

**FIGURE 1:**
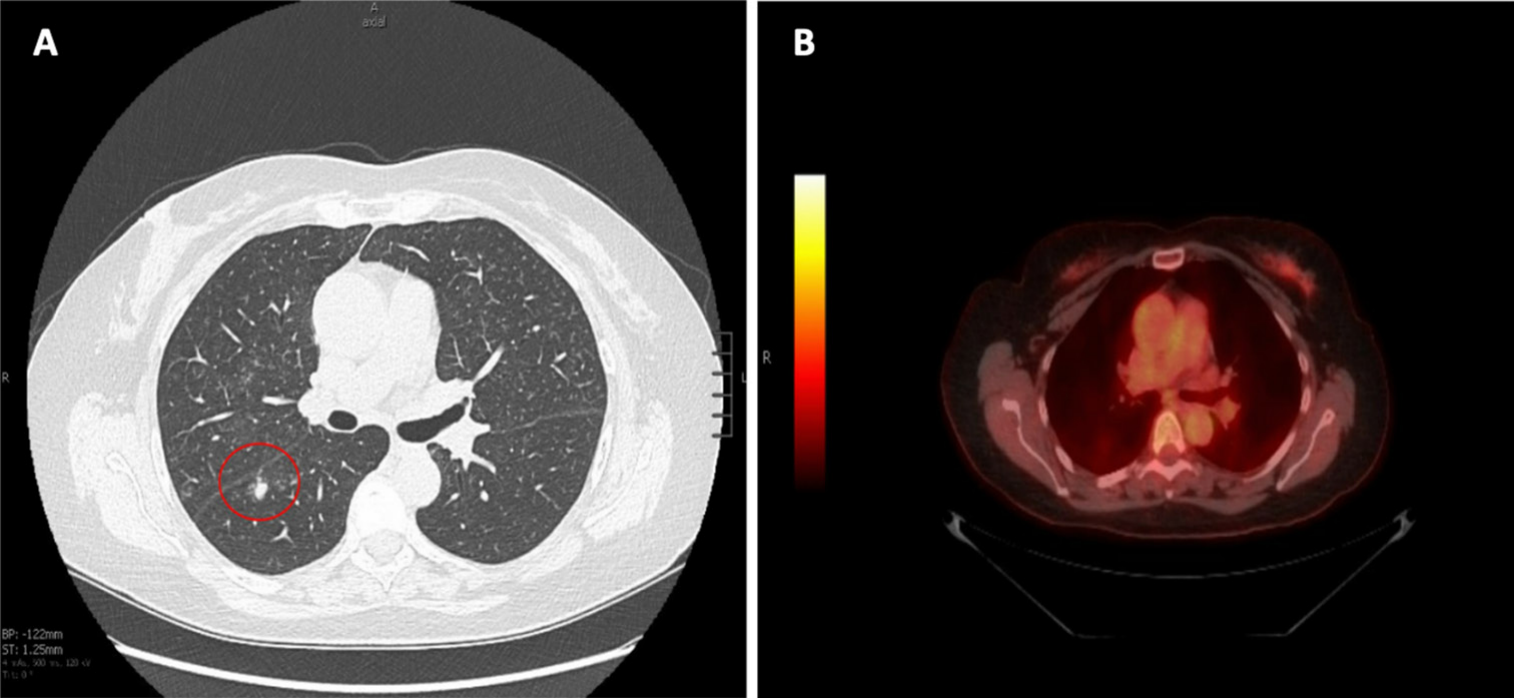
(A) Initial computed tomography (CT) scan of chest (axial view) showing faint upper lobe ground glass opacities and prominent right lower lobe (RLL) solid 6 mm nodule with surrounding ground glass; no lymphadenopathy seen. (B) Positron emission tomography (PET) scan with no fluorodeoxyglucose uptake at site of lesions.

A 64‐year‐old female nonsmoker with a history of well‐controlled systemic lupus erythematosus (SLE) was referred to the pulmonary clinic for an incidental lung finding. A routine chest computed tomography (CT), performed to evaluate for coronary calcification, revealed multiple solid and subsolid pulmonary nodules, with the most prominent in the right lower lobe (RLL) at 6 mm (Figure 1A). The patient denied any respiratory or systemic symptoms including cough, dyspnea, hemoptysis, arthralgia, skin changes, or weight loss. Pulmonary function tests, infectious disease workup and inflammatory markers were within normal limits. Antinuclear antibody and double‐stranded DNA titers were mildly elevated; however, the remainder of the rheumatologic workup was unremarkable.

A subsequent Positron emission tomography (PET) scan demonstrated no abnormal fluorodeoxyglucose uptake, including in the bilateral pulmonary nodules (Figure 1B). Over a 2‐year period, serial CT imaging showed interval growth of the RLL subsolid nodule from 6 to 9 mm, without evidence of lymphadenopathy. Given this growth, the patient underwent percutaneous needle biopsy of the RLL lesion, which revealed pulmonary MALT lymphoma. As the patient was asymptomatic, a watchful waiting approach was taken.

Surveillance imaging over 2 more years identified a new 8 mm subsolid nodule in the right middle lobe (RML) along with more diffuse ground‐glass opacities (GGO). Concern for a secondary process prompted video‐assisted thoracoscopic surgery biopsy of the RML nodule, which again confirmed pulmonary MALT lymphoma. Given the diffuse radiographic findings, the patient was initiated on treatment with a course of rituximab. Simultaneously, hydroxychloroquine was started for arthralgia related to her underlying SLE.

**FIGURE 2:**
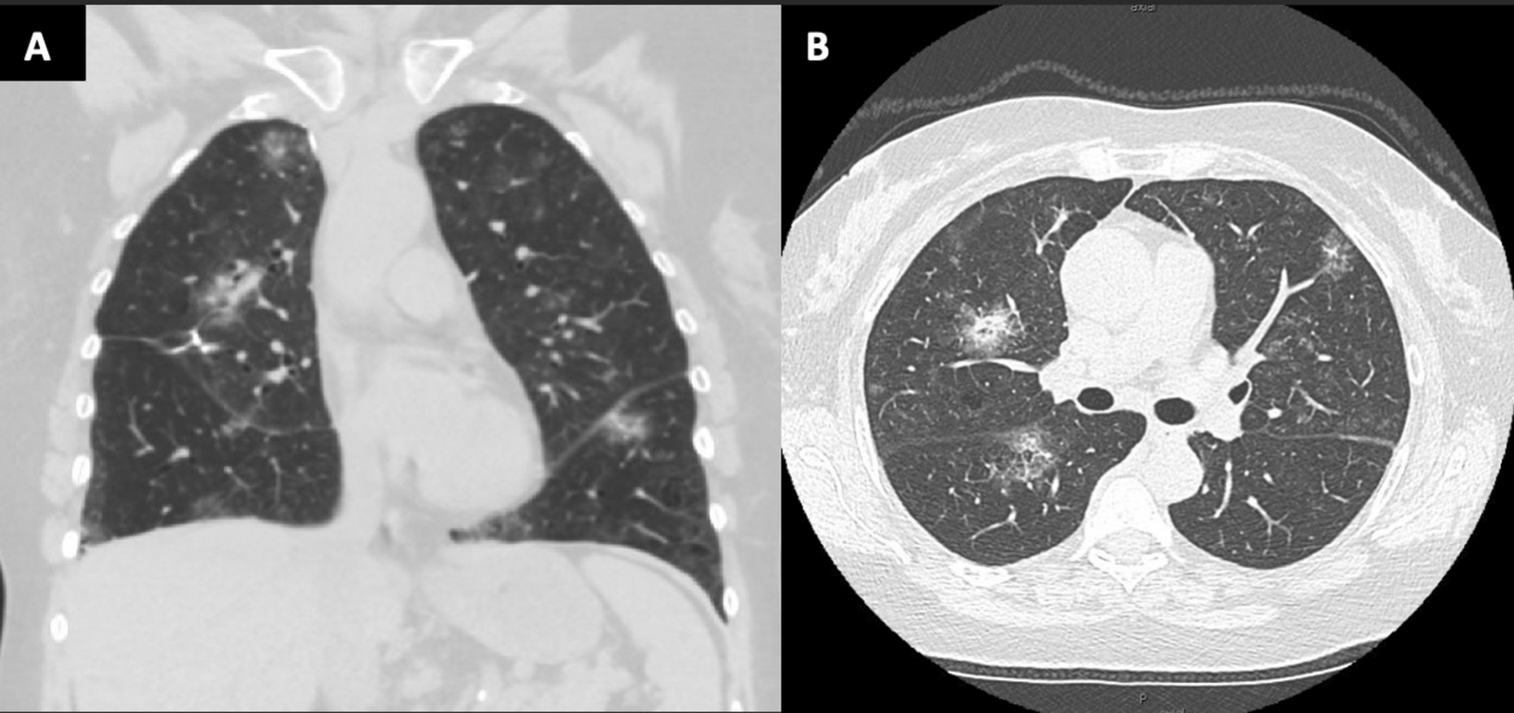
(A) Coronal view computed tomography (CT) of the chest shows waxing and waning nodules with an 8 mm semisolid right middle lobe (RML) lesion and increased bilateral ground glass opacities one year after rituximab treatment and two years after the initial diagnosis. (B) Axial view chest CT scan.

One year after completion of rituximab, mild radiographic improvement was observed, with persistent waxing and waning pulmonary nodules (Figure 2). A subsequent bone marrow biopsy confirmed the absence of tumour infiltration. The patient has remained without pulmonary symptoms throughout her 5 years since diagnosis and continues under surveillance, with consideration of another course of rituximab or combined rituximab‐bendamustine should radiographic progression occur.

## 3 Discussion

MALT lymphomas arise from uncontrolled proliferation of mucosal B cells, with a median age at the time of diagnosis between 50 and 60 years old. The survival rate of patients with pulmonary MALT lymphoma is as high as 85%–95% at 5 years. Although the prognosis is favourable, given the indolent course and variable clinical presentation of pulmonary MALT lymphoma, diagnosis can be challenging.

No specific signs or symptoms are pathognomonic, and approximately 36% of patients are asymptomatic at diagnosis. Although CT findings are also typically nonspecific, retrospective analyses have identified certain patterns. Deng et al. reported that bilateral consolidations with air bronchograms were the most frequent presentation, followed by bilateral nodules or masses [2]. Rarer manifestations included bilateral GGOs, reticular patterns, unilateral lesions and mediastinal lymphadenopathy. In our case, the predominant finding of diffuse GGOs highlights the wide spectrum of possible imaging features, which can closely mimic benign inflammatory or infectious lung diseases and thereby obscure diagnosis. PET‐CT, as in our case, frequently demonstrates minimal or absent uptake, further complicating recognition. Definitive diagnosis therefore requires histopathologic confirmation.

Another important feature is the association with autoimmune conditions. Previous reports have most often described links with Sjögren's syndrome, systemic sclerosis and lymphoid interstitial pneumonia. Reports of pulmonary MALT lymphoma in patients with SLE are exceedingly rare, and our case adds to the growing evidence that chronic autoimmune‐mediated inflammation may play a role in lymphomagenesis.

Because of the disease's rarity, no randomised controlled trials have been conducted to establish an optimal treatment approach. Given the generally favourable prognosis and relatively asymptomatic course, a watch and wait strategy is often reasonable. For symptomatic, localised disease, surgical resection or radiotherapy can provide local control, whereas unresectable or disseminated disease is typically managed with anti‐CD20 monoclonal antibody therapy (rituximab) or systemic chemotherapy. Limited evidence suggests that macrolide antibiotics, particularly clarithromycin, may also induce partial or complete remission in some cases of pulmonary MALT lymphoma, potentially via direct antineoplastic and immunomodulatory effects independent of antimicrobial activity [3]. No studies to date have specifically evaluated the treatment of an underlying autoimmune process and its role in disease remission [4]. In this patient, therapy for SLE combined with a course of rituximab produced modest radiographic improvement. The persistence of disease despite immunosuppression raises questions about the interplay between autoimmune control and lymphoma behaviour. Therefore, further multicentre prospective studies are needed to clarify the role of macrolides such as clarithromycin in pulmonary MALT lymphoma, as well as to determine whether targeted treatment of the underlying autoimmune process can improve radiographic and clinical outcomes.

This case illustrates the chronic indolent course of pulmonary MALT lymphoma, highlights its capacity to mimic other diseases, especially radiographically and underscores its association with SLE, supporting the concept that chronic inflammation can drive lymphoproliferative disease.

## Author Contributions

Rajan Patel conceived and wrote the first draft of the case report, conducted the literature review and prepared the final submission. Diane Stover, as the principal investigator, critically revised the manuscript for important intellectual content and provided overall supervision. Both authors reviewed and approved the final version of the manuscript.

## Consent

The authors declare that written informed consent was obtained for the publication of this manuscript and accompanying images and attest that the form used to obtain consent from the patient complies with the Journal requirements as outlined in the author guidelines.

## Conflicts of Interest

The authors declare no conflicts of interest.

## Data Availability

Data sharing not applicable to this article as no datasets were generated or analysed during the current study.
